# Interrogation of γ-tubulin alleles using high-resolution fitness measurements reveals a distinct cytoplasmic function in spindle alignment

**DOI:** 10.1038/s41598-017-11789-7

**Published:** 2017-09-12

**Authors:** Kristian Shulist, Eric Yen, Susanne Kaitna, Allen Leary, Alexandra Decterov, Debarun Gupta, Jackie Vogel

**Affiliations:** Department of Biology, McGill University, 3649 Promenade Sir William Osler, Montreal, Quebec, H3G 0B1 Canada

## Abstract

γ-Tubulin has a well-established role in nucleating the assembly of microtubules, yet how phosphorylation regulates its activity remains unclear. Here, we use a time-resolved, fitness-based SGA approach to compare two γ-tubulin alleles, and find that the genetic interaction profile of γtub-Y362E is enriched in spindle positioning and cell polarity genes relative to that of γtub-Y445D, which is enriched in genes involved in spindle assembly and stability. In γtub-Y362E cells, we find a defect in spindle alignment and an increase in the number of astral microtubules at both spindle poles. Our results suggest that the γtub-Y362E allele is a separation-of-function mutation that reveals a role for γ-tubulin phospho-regulation in spindle alignment. We propose that phosphorylation of the evolutionarily conserved Y362 residue of budding yeast γ-tubulin contributes to regulating the number of astral microtubules associated with spindle poles, and promoting efficient pre-anaphase spindle alignment.

## Introduction

**Figure 1:**
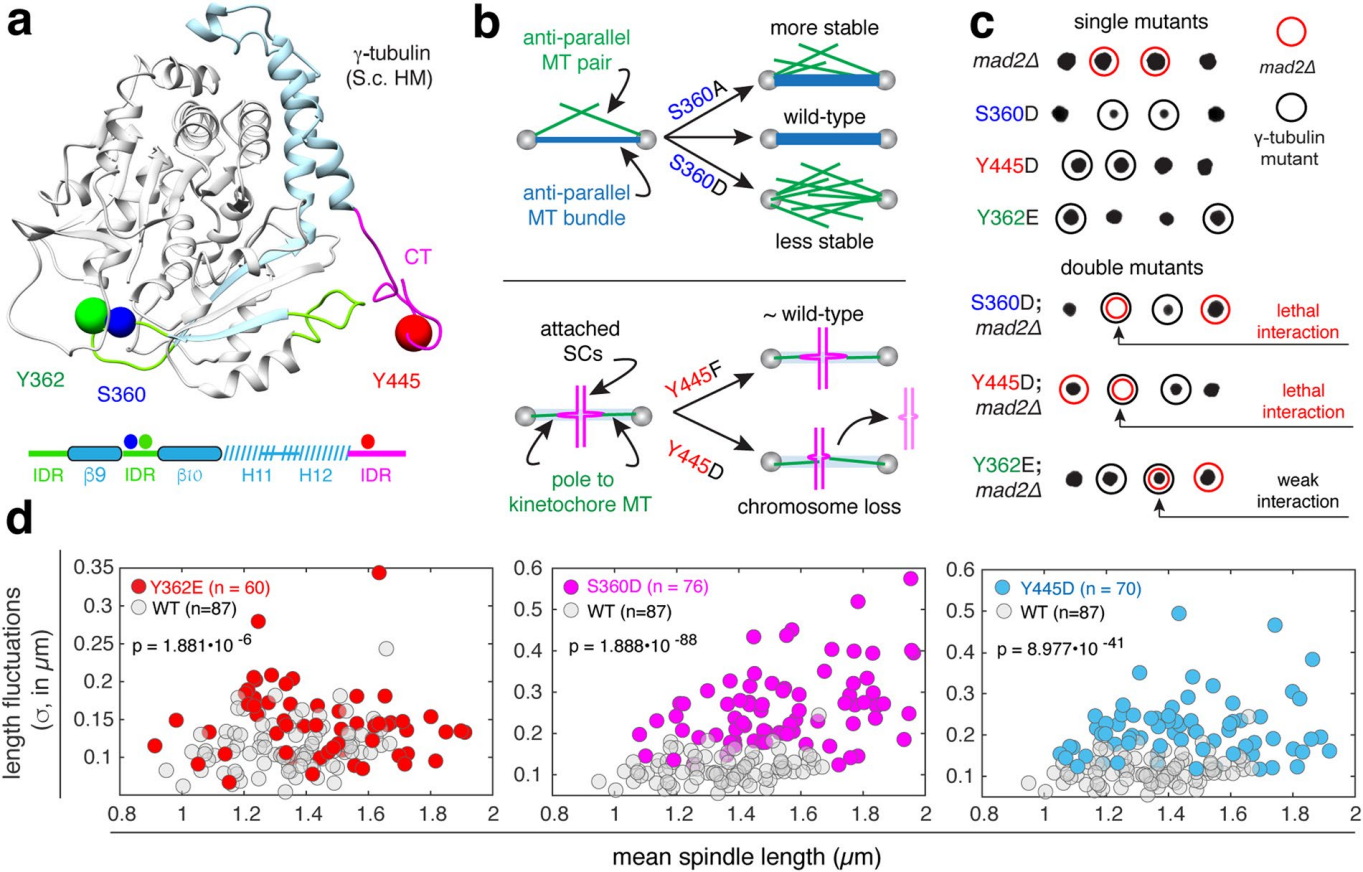
Effects of γ-tubulin phosphomimetic (D/E) mutations on spindle function and spindle stability. (**a**) ribbon structure of yeast γ-tubulin (homology model based on the human γ-tubulin structure) with loops and structured domains in the final 132 residues shown in green and blue, the predicted intrinsically disordered (ID) c-terminus is shown in pink. Spheres (0.25 nm) indicate the positions of the side chains of residues known to be phosphorylated at SPBs *in vivo*; S360 (blue), Y362 (green) and Y445 (red). A schematic representing the primary sequence of the 132 residues is shown below with ID regions shown in green, the α-helices and β-strands shown in dashed blue and solid blue (respectively), and the C-terminus shown in pink. (**b**) Cartoon depicting the role of S360 in architecture of the anti-parallel microtubule (MT) bundle and spindle stability (left panel); schematic depicting the role of Y445 in the attachment of sister chromatids (SCs) and resulting chromosome loss (right panel). (**c**) Tetrad dissections of γtub-S360D, γtub-Y445D and γtub-Y362E, alone and in combination with mad2Δ. Unlike γtub-S360D and γtub-Y445D, the γtub-Y362E mad2∆ double mutant is viable, with a slight growth defect, at 25 °C. (**d**) Spindle length fluctuations in wild-type, γtub-Y362E, γtub-S360D and γtub-Y445D cells, plotted as a function of mean spindle length. Fluctuations for each spindle (σ) are computed as the standard deviation of length.

γ-Tubulin is an evolutionarily conserved member of the tubulin superfamily^1,2^ whose essential function is the nucleation of microtubules (MTs) in eukaryotic cells^2–7^. In budding yeast, γ-tubulin is encoded by the essential gene *TUB4*^8^. γ-Tubulin, Spc97 and Spc98 form γ-tubulin complexes (γTuCs) located on the nuclear and cytoplasmic surfaces of spindle pole bodies (SPBs), organelles that serve as the centrosome in yeast cells and remain embedded in the nuclear envelope throughout mitosis^9–11^. The γTuC nucleates both spindle (nuclear) and astral (cytoplasmic) MTs^12–14^ and γ-tubulin bound to SPBs has been proposed to be in the form of “active” (nucleation competent) γTuCs^15^. The pool of γ-tubulin bound to purified SPBs is phosphorylated at eight residues: two (T130 and T227) are specific to cells arrested in G1 and six (S42, S43/T44, S360, Y362, S444 and Y445) are specific to cells arrested in metaphase^16^. Residues S360, Y362 and Y445 (Fig. 1a) are evolutionarily conserved^16,17^. Phosphorylation of residues S360, Y362 and Y445 occurs during metaphase and before the anaphase transition^16^, a time in the cell cycle that is functionally relevant to spindle assembly and sister chromatid attachment^18^.

The conservation of S360, Y362 and Y445 between yeast and human^16^ suggests phospho-regulation of these residues is a general mechanism for controlling the activity of γ-tubulin. To date, there is no evidence that phosphorylation of the pool of γ-tubulin bound to the SPBs contributes to the activation of γTuCs^15^. Phosphorylation of S360 and Y445 likely contributes to events in spindle assembly (Fig. 1b). Mutation of S360 alters the number of anti-parallel MTs that provide stability to the spindle; an alanine substitution at this residue increases the number of anti-parallel MTs and stabilizes the spindle, while substitutions such as aspartic or glutamic acid that introduce a local negative charge and mimic constitutive phosphorylation, block the formation of anti-parallel MTs and decreases spindle stability^19^. Mutation of Y445 disrupts spindle function^17^ and causes chromosome loss^20^; as a result, Y445D cells cannot survive in the absence of the spindle assembly checkpoint (SAC)^17,21,22^. An internal deletion of S444, Y445 and their adjacent residues (DSYL) within the intrinsically disordered C-terminus perturbs both spindle assembly and pre-anaphase spindle positioning^23,24^. As with S360 and Y445, Y362 is also evolutionarily conserved, however the biological function of this modification is unknown.

Separation-of-function mutations are extremely useful genetic tools in cell biology, and aid in understanding the biological significance of γ-tubulin phospho-regulation. Separation-of-function mutations can be identified through the analysis of synthetic genetic interactions (SGIs) discovered by conducting synthetic genetic array (SGA) screens^25^. The SGA screen is a robotic-assisted method used to assess genetic interactions of mutations arrayed on plates; typically the array consists of non-essential gene knock-outs^26–29^. The relative growth of each strain (wild-type controls, query, array and double mutants) is extracted using end-point analysis of colony size, with the growth of the double mutant compared to that of each single mutant; if the double mutant has an unexpected phenotype, an SGI is inferred. For null or loss-of-function mutations, the SGI profiles of functionally redundant genes converge, with genes in one pathway showing strong congruence in interactions with those in parallel, redundant pathways^29^. However, a subset of unique SGIs is expected in the case of separation-of-function mutations when comparing SGI profiles for a series of alleles. Analysis of alleles presents an additional challenge, as they may exhibit phenotypic differences dependent on the process that is perturbed by the mutation. For example, an allele with a mutation that perturbs one of two spindle positioning pathways may exhibit a weak phenotype in comparison to an allele with a mutation that perturbs chromosome attachment. An SGA-based approach for detection of separation-of-function mutations must therefore be sensitive to differences in relative fitness of both single and double mutants, and also be precise in measurements such that SGI profiles for multiple alleles of the same gene can be compared.

In this study, we describe GAMER (Genetic Array with Mixed Effects Regression), a time-resolved and fitness-based SGA method, and used this approach to perform a comparative functional analysis by extracting SGIs of two γ-tubulin alleles (γtub-Y362E and γtub-Y445D) used as query mutations in combination with ~4,700 SGA deletion mutations. Growth rates were computed and used to calculate the fitness of wild-type controls, and query, array and double mutants, and to compare relative fitness of mutants across the two γ-tubulin query alleles. The SGI profile for the previously described γtub-Y445D mutation^17^ was enriched for genes that are involved in spindle assembly and stability, act in chromosome segregation, or function in the SAC, monitoring the fidelity of sister chromatid attachment. The SGI profile for the γtub-Y362E mutation showed convergence with the γtub-Y445D mutant for a subset of genes involved in mitosis, but also had a distinct set of SGIs with genes acting in spindle positioning and alignment relative to the future plane of cell division, as well as cell polarity. Live cell analysis of spindle stability and movements relative to the bud neck and polarity axis revealed that the γtub-Y362E mutation does not alter spindle stability but is defective in pre-anaphase spindle alignment. We report that in the γtub-Y362E mutants, the number of astral MTs is increased, and the bias of astral MTs to the old SPB relative to the new SPB is lost, leading to inefficient spindle alignment. Our results provide new evidence that the phosphorylation of an evolutionarily conserved tyrosine residue (Y362) contributes to γ-tubulin function in controlling the number of astral MTs associated with both SPBs of the metaphase spindle, and influences the efficiency of pre-anaphase spindle alignment relative to the future plane of cytokinesis.

## Results

### The γtub-Y362E mutation does not reduce spindle stability

Cells with defects in spindle function require the activity of the SAC for survival, and typically die in the absence of checkpoint proteins^22^. The viability of tub4 alleles and their dependence on SAC activity was tested by analysis of progeny (tetrads; 20 per strain) obtained from diploids heterozygous for γ-tubulin alleles γtub-S360D, γtub-Y445D or γtub-Y362E and the mad2∆ null mutation which blocks SAC activation. γ-Tubulin mutations S360D, Y445D and Y362E are viable under normal growth conditions (25–30 °C), with a weak growth defect observed for γtub-S360D and γtub-Y445D (^16,17^ and Fig. 1c). As previously shown, the γtub-S360D and γtub-Y445D mutations are lethal in combination with the mad2∆ mutation^16,17^ while the Y362E mutant did not show similar sensitivity to the loss of the SAC (Fig. 1d).

We next examined fluctuations in spindle length, which reports relative spindle stability^19^. Length fluctuations were computed from the standard deviation of spindle length (σ) and assessed with respect to the mean spindle length (Fig. 1e). The γtub-S360D mutation disrupts the formation of inter-polar anti-parallel cross-linked MTs^19^, and spindles in γtub-S360D cells are unstable and undergo very large length fluctuations relative to wild-type cells (p = 1.888 × 10^−85^). In γtub-Y445D cells, spindles experience large length fluctuations relative to wild-type cells (p = 8.977 × 10^−41^). The length fluctuations of spindles in γtub-Y362E cells are similar to those of wild-type spindles (p = 1.881 × 10^−6^), and both wild-type and γtub-Y362E spindles are stable relative to the spindles of γtub-S360D and γtub-Y445D mutants.

### GAMER analysis reveals common and distinct SGIs of γtub-Y362E and γtub-Y445D alleles

**Figure 2:**
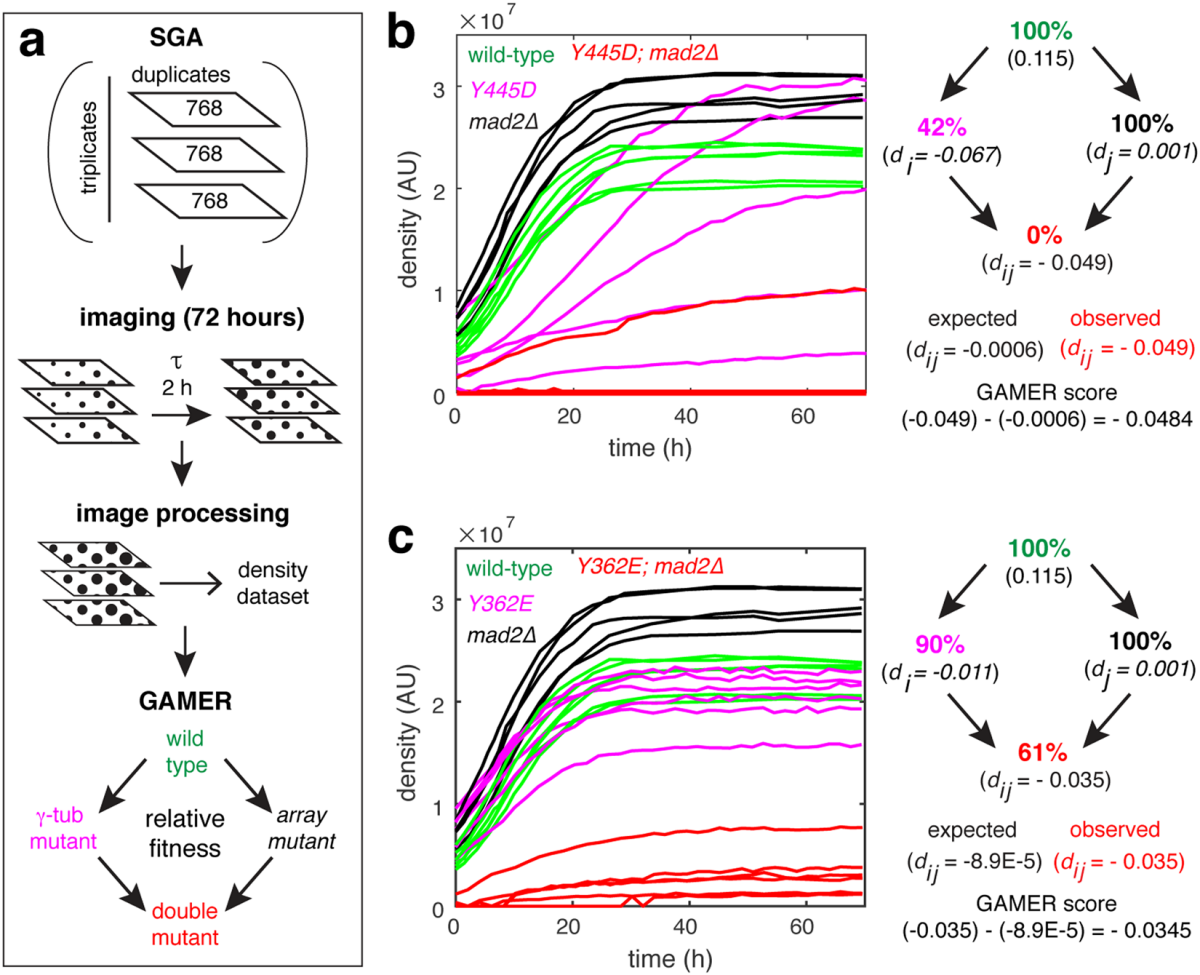
Identification of synthetic genetic interactions based on relative fitness using GAMER. (**a**) Overview of the GAMER method, which includes imaging every two hours over a 72-hour period. Images are processed computationally and fitness extracted and used to calculate the GAMER scores of the double mutants. (**b**,**c**) SGA growth curves (left panels) for six isolates of wild-type, γtub-Y445D (top) or γtub-Y362E (bottom), mad2Δ and the combined double mutant. Values of the observed and expected growth defects (d_ij_) for the respective double mutants (right panels).

GAMER analysis uses a modified SGA method that provides a growth rate parameter. After the final pinning for selection of *MATa* double mutants, plates were imaged at two hour intervals for 72 hours (movie M1). Colony size and density were extracted from the resulting images and used to calculate growth rate and relative fitness of each strain (Fig. 2a). Linkage group analysis for the *TUB4* locus extending 150 kilobases in both directions (approximately 50 centiMorgans^30–32^) was conducted using both GAMER and a standard growth characterization method (Supplemental Figure S1). Due to technical issues arising from experimental procedures (i.e. colony position on the array plate), growth characterization of the endpoint colony sizes had much larger variances. These issues can be corrected during post-processing^33^. In contrast, the resulting GAMER growth rates for the *TUB4* linkage groups had a higher measured precision and showed more uniformity across the genes without additional post-processing.

**Figure 3:**
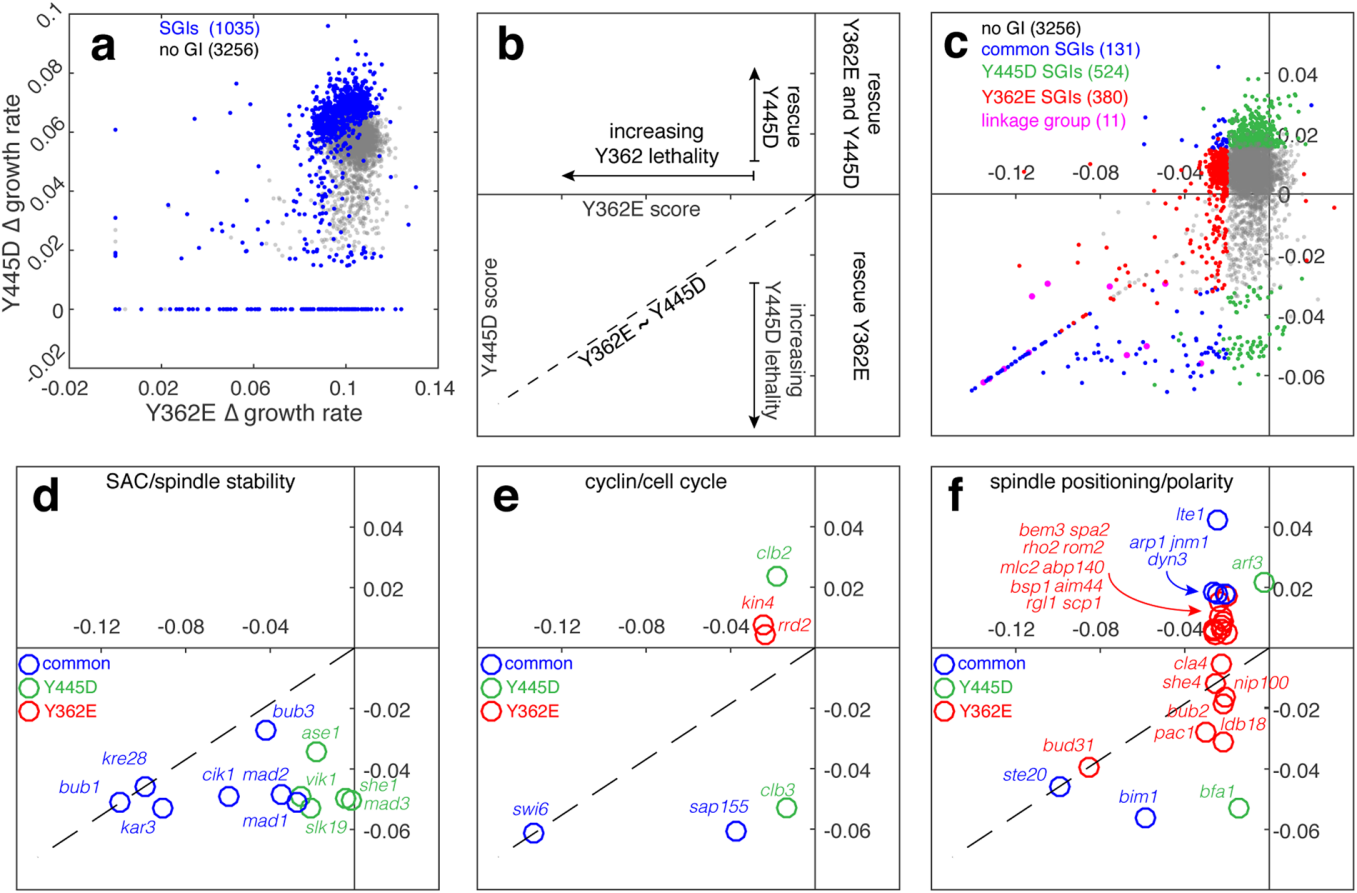
GAMER identifies distinct and common SGIs for γtub-Y445D and γtub-Y362E alleles. (**a**) Growth rates for all array mutants when combined with γtub-Y445D were plotted against their growth rates when combined with γtub-Y362E. The blue points represent all significant hits between the two GAMERs; grey represents the genes with no genetic interactions. Genes that caused lethal phenotypes when combined with γtub-Y445D or γtub-Y362E cluster along the x- or y-axis, respectively. (**b**) The resulting GAMER scores for γtub-Y445D and γtub-Y362E were projected on the same axis organizing SGIs into four quadrants depending on the respective scores: [i] hits that reduced the relative fitness in both alleles (lower, left), [ii] hits that reduced fitness for γtub-Y362E, but increased fitness (rescued) for γtub-Y445D (upper, left), [iii] hits that increased fitness for γtub-Y362E but reduced fitness for γtub-Y445D (lower, right), and [iv] hits that increased fitness for both alleles (upper, right). Quadrant [i] is divided in half along a diagonal line (—) which indicates hits that reduced fitness similarly for both alleles. Above this line, fitness was reduced more for γtub-Y362E than γtub-Y445D while below this line, the opposite is true. (**c**) GAMER scores for all SGA mutants including those that were unique hits for γtub-Y445D (green), those that were unique hits for γtub-Y362E (red), those that were common hits for both alleles (blue), the linkage group (pink) and those that did not have SGIs with either allele (grey). (**d**–**f**) SGIs related to the SAC and spindle stability (**d**), to the cell cycle (**e**), and to spindle positioning and cell polarity (**f**).

In agreement with the mad2∆ genetic analysis shown in Fig. 1c, γtub-Y445D was found to have a strong synthetically lethal interaction with mad2∆ (relative fitness of 0%, p < 10^−4^, Fig. 2b). GAMER is also suitable for detection of weak SGIs; the γtub-Y362E mutation had a weak but significant interaction with mad2∆ (relative fitness of 61%, p = 8.88 × 10^−3^, Fig. 2c) revealing an interaction that was not apparent when colony sizes of γtub-Y362E and mad2∆ were compared with the double mutant (Fig. 1c). Fitness-based experiments using GAMER overcome potential technical variations that could influence the interpretation of results; for example mad2∆ colonies appear to increase in size faster than wild-type due to their increased endpoint densities (Fig. 2b,c; left panels), however, our analysis reveals that the growth rates of mad2∆ and wild-type colonies are not statistically different (Fig. 3B,C).

GAMER identified 1035 SGIs across the γtub-Y362E and γtub-Y445D screens (Fig. 3a and supplemental dataset 1). To directly compare the relative fitness between SGIs of the two alleles, we projected the two sets of GAMER scores (γtub-Y362E and γtub-Y445D) for each array mutant. This organized SGIs into groups: rescues, where the fitness of the γ-tubulin allele (Y445D) was increased as a result of the array mutant, or the fitness of the array mutant increased when combined with the γtub-Y362E or γtub-Y445D allele, or synthetic sick or lethal SGIs where the fitness of the γ-tubulin allele combined with the array mutant was reduced. Genes in the linkage group (Fig. 3c, pink) tend to cluster close to the boundary (—) between where one γ-tubulin allele is reduced more than the other (i.e. where fitness is equivalently reduced for both γtub-Y362E and γtub-Y445D).

Comparison of the GAMER-derived SGIs of γtub-Y362E and γtub-Y445D mutants revealed that the proportion of SGIs common to both alleles was smaller than the set of SGIs unique to each allele. We identified 123 SGIs in common for γtub-Y445D and γtub-Y362E, and a larger number of unique SGIs, with 524 SGIs for γtub-Y445D and 380 SGIs for γtub-Y362E. γTub-Y362E did not have an SGI with the mad3Δ mutation and the normalized scores for the γtub-Y362E interactions with mad1Δ and mad2Δ mutations were less severe than the corresponding scores for γtub-Y445D (Fig. 3c and supplemental datasets). The γtub-Y362E mutation had no SGIs with mutations in genes that strictly contribute to the function of spindle/nuclear microtubules (Fig. 3d). In contrast, SGIs specific for the γtub-Y445D allele were consistent with defects in both anti-parallel (ase1Δ and slk19Δ) and parallel (vik1∆) nuclear MT cross-linking, the former is required to form the interpolar MTs that stabilizes the spindle^34–37^. SGIs common to both alleles were often genes that function in both spindle MT and astral MT-dependent processes or coordinate the completion of spindle positioning/alignment with mitotic exit. Cik1∆, which has been shown to function on both the spindle and astral MTs^38^, was a common SGI for both alleles. The γtub-Y362E SGI profile revealed a dependence on the cell cycle kinase Kin4 (Fig. 3e), which inhibits the mitotic exit network (MEN) when the spindle positioning checkpoint is activated^39,40^. Additionally, γtub-Y362E SGI profile was enriched for SGIs with genes acting in Kar9 and dynein-dependent spindle positioning and cell polarity (Fig. 3f). This group of SGIs included mutations in bub2∆ and lte1∆, as well as mutations of components of the dynactin complex (arp1Δ, jnm1∆, ldb18∆, nip100∆) and the kinase Pac1, both of which regulate the activity of cytoplasmic dynein during spindle positioning. SGIs specific to γtub-Y362E also included components of the polarisome (rho2∆, bem3∆, rom2∆, rgl1∆) and actin cables (abp140∆) that are critical for both Kar9 and dynein dependent spindle positioning. These results suggested that the γtub-Y362E mutation likely perturbs a function of γ-tubulin that is directly linked to spindle positioning and actin organization rather than the previously characterized role in spindle assembly (γtub-S360D, γtub-Y445D).

Despite their spatial separation in both the primary and tertiary structures of γ-tubulin, the γtub-S360D and γtub-Y445D mutants both exhibit spindle instability (Fig. 1a). Residues S360 and Y362 are positioned within an unstructured loop, close to each other in the primary and tertiary structure of γ-tubulin and on the opposite surface of the molecule to the intrinsically disordered C-terminal containing Y445. We asked if S360 and Y362 are functionally coupled with Y445 by assessing the viability of intra-molecular double mutants in S360 and Y445 as well as in Y362 and Y445. Individual phospho-inhibiting mutations (A or F) in S360, Y362 and Y445 were viable (Supplemental Figure S2a). Interestingly, intra-molecular double mutations S360A-Y445D or S360D-Y445F were lethal (Supplemental Figure S2b). Previously we reported that S360 phosphorylation by Cdk1 requires the early mitotic cyclin Clb3 *in vivo*^41^, and this may be the cause of the lethal interaction between the γtub-Y445D and clb3∆ mutants (Fig. 3e). When mutations in Y362 and Y445 were combined, only the Y362F-Y445D double mutant showed a growth phenotype (Supplemental Figure S2c). Taken together with the GAMER results, our analysis of intramolecular genetic interactions suggests that phosphorylation of S360 and Y445 must be coordinated with respect to each other during spindle assembly, while phosphorylation of Y362 is uncoupled and regulates γ-tubulin activity in a process that involves genes acting in spindle alignment and cell polarity (Supplemental Figure S2d).

### A geometric definition for perfect alignment of the metaphase spindle in budding yeast

**Figure 4:**
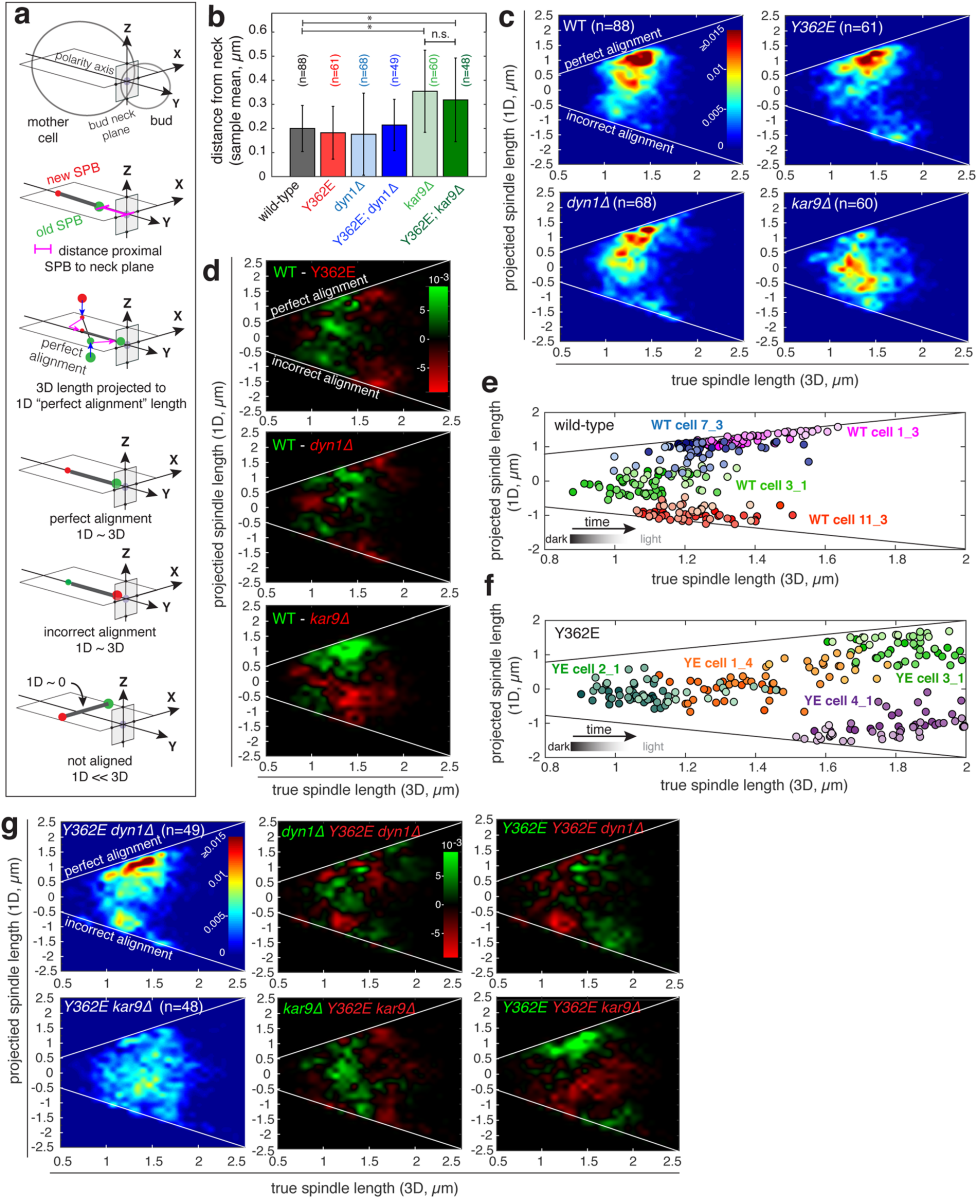
Pre-anaphase spindle alignment is perturbed in γtub-Y362E cells. (**a**) Schematic depicting the method used to compute the distance between proximal pole and bud neck (spindle positioning) and spindle alignment in relation to “perfect alignment”. Perfect alignment is defined as a spindle with a 1D projected spindle length ~3D spindle length. (**b**) Distance of the proximal pole from the bud neck; error bars show standard deviation. *Indicates statistical difference with p < 0.0001; n.s. denotes no significant difference. (**c**) Heatmaps showing the projected 1D spindle length as a function of true 3D spindle length in wild-type (WT), γtub-Y362E, dyn1∆ and kar9∆ cells. Color bar shows the frequency of time points normalized to the total number of time points of all cells. Incorrect alignment occurs when the new pole is proximal to the bud. (**d**) Difference map comparing WT (shown in (**c**); upper left) to γtub-Y362E (top), dyn1∆ (middle) and kar9∆ (bottom). Green indicates areas where the frequency of the WT was increased relative to the mutant, red indicates areas where the frequency of the mutant was increased relative to WT. Instantaneous projected 1D spindle length plotted as a function of true 3D spindle length of representative (**e**) WT and (**f**) γtub-Y362E cells. Time is indicated by the color gradation from dark (start) to light (end). (**g**) The γtub-Y362E mutation increases the range of unaligned spindle orientations and incorrect alignment when combined with kar9∆ and dyn1∆ mutants. Heatmaps showing the projected 1D spindle length in double mutants γtub-Y362E; dyn1∆ (upper left) and γtub-Y362E; kar9∆ cells (lower left). Plots showing the differences between the single mutants and double mutants. Green indicates areas where the frequencies of the single mutant were larger than the double mutants, red indicates areas where the frequencies of the double mutant were increased.

A budding yeast cell is asymmetric once the bud has formed, with a polarity axis that is normal to the plane of the bud neck (the future site of cytokinesis). As the spindle is assembled, it becomes positioned close to the bud neck and is aligned parallel to the polarity axis and normal to the bud neck plane, with a strong bias for the older SPB (formed in the previous cell cycle) to be proximal to the neck^42^. This condition, which we define as perfect alignment, is the reference for our measurement of spindle alignment in wild-type and mutant cells (Fig. 4a). Perfect alignment ensures that the anaphase spindle will elongate through the bud neck and a full complement of chromosomes are inherited by the mother and daughter cells at the end of cell division. If the spindle is not positioned at the neck and/or not aligned normal to the bud neck plane, anaphase may occur in the mother cell, resulting in aneuploidy and cell death. To compare a spindle to this reference, we project it (in 3D) to a perfectly aligned spindle (1D). If the spindle is perfectly aligned, the true (3D) and projected (1D) lengths are approximately the same, while spindles rotated 90° off the polarity axis would have a projected length of 0. As spindle alignment proceeds, the true and projected length of the spindle are expected to converge.

### The γtub-Y362E mutation does not perturb spindle positioning at the bud neck

We measured spindle positioning at the bud neck in wild-type and γtub-Y362E cells, as well as in mutants lacking either cytoplasmic dynein (dyn1∆) or the APC-like + tip protein, Kar9 (kar9∆), both of which are central to the two functionally redundant spindle positioning pathways in budding yeast. To measure spindle position relative to the bud neck, we analyzed the trajectory of the SPBs, which are diffraction limited point-like objects whose position can be precisely determined^19,43^, over a ten-minute window in single cells and extracted the mean distance of the proximal SPB to the neck plane and the mean spindle length using 3-dimensional data collected at ten second intervals. The distance of the proximal SPB to the neck plane was computed for metaphase spindles with an average length <2 µm and initial lengths between 0.8 µm and 2 µm for wild-type (n = 88 cells), single mutant (γtub-Y362E n = 61 cells; kar9∆ n = 60 cells; dyn1∆ n = 68 cells) and double mutant strains (γtub-Y362E kar9∆ n = 48 cells*;* γtub-Y362E dyn1∆ n = 49 cells). In the majority of wild-type, γtub-Y362E, dyn1∆ and γtub-Y362E dyn1∆ cells, the normalized mean distance of the proximal pole to the neck is less than 25% of the diameter of the mother cell regardless of the mean length of the spindle (Fig. 4b and Supplemental Figure S3). In contrast, in the majority of kar9∆ and γtub-Y362E kar9∆ cells, the mean distance of the proximal pole to the neck is >25% of the diameter of the mother cell and is significantly increased relative to wild-type, as well as to the other mutants (p < 0.0001; Fig. 4b and Supplemental Figure S3). Movement of the spindle to the neck occurs even in the absence of cytoplasmic dynein, and is not significantly perturbed by the γtub-Y362E mutation.

### Spindle alignment is defective in the γtub-Y362E mutant

For each strain (wild-type or mutant), alignment is shown as the normalized frequency of projected (1D) lengths in a distribution of 3D length bins for all time points; two white lines indicates where 1D and 3D length converge, either perfect alignment (aligned, old SPB proximal to neck) or incorrect alignment (aligned, new SPB proximal to neck). In wild-type cells, perfect alignment is correlated with increasing 3-dimensional spindle length, with most spindles ≥1.5 µm in length maintaining perfect alignment (Fig. 4c, upper left panel). In the γtub-Y362E mutant, spindles exhibit a defect in achieving or maintaining perfect alignment, and the frequency of incorrect alignment of spindles >1.5 µm in length is increased relative to wild-type (Fig. 4c, upper right panel). Spindle alignment is observed in dyn1∆ cells (Fig. 4c, lower left panel), however the majority of spindles in kar9∆ cells do not achieve or maintain perfect alignment regardless of spindle length (Fig. 4c lower right panel). Instead, spindles in kar9∆ cells tend to explore orientations centered around a parallel alignment to the bud neck and 90° to the polarity axis (i.e. projected 1D length of zero). We directly compared spindle alignment in wild-type cells with mutants by computing the difference between the conditions, with wild-type compared to γtub-Y362E, dyn1∆ or kar9∆ (Fig. 4d). Spindles in γtub-Y362E cells explore many orientations relative to the bud neck plane. The frequency of incorrect alignment is increased in both γtub-Y362E cells, and to a lesser amount in dyn1∆ cells, relative to wild-type cells.

To better understand the nature of the defect in spindle alignment in γtub-Y362E cells, we plotted instantaneous projected spindle length as a function of true spindle length for representative wild-type and γtub-Y362E cells taken from mean 3D length bins of 1 µm, 1.3 µm and 1.5 µm (Fig. 4e,f). In wild-type cells, spindles with an average length less than 1.2 µm are not aligned, however longer spindles either undergo alignment or remain aligned during the ten minutes of observation (Fig. 4e and supplemental movies M2–M5). In contrast, in γtub-Y362E cells, spindles are frequently misaligned independent of spindle length, and approach but do not maintain perfect alignment as spindle length increases (Fig. 4f, and supplemental movies M6–M9).

### The γtub-Y362E mutation enhances alignment defects of Kar9 and dynein mutants

To understand how the γtub-Y362E mutation might affect the function of Kar9 or dynein, we next investigated spindle alignment in γtub-Y362E cells in combination with dyn1∆ or kar9∆ mutations. When γtub-Y362E is combined with dyn1∆, such that spindle alignment is dependent on Kar9, the frequency of incorrect alignment increases for spindles ≤1.5 μm (Fig. 4g, upper panels). Interestingly, while the majority of spindles in kar9∆ cells were biased to orientations parallel to the bud neck axis (i.e. projected 1D length of zero; Fig. 4c), in γtub-Y362E kar9∆ double mutants, spindle orientation appears to be random relative to the bud neck plane (Fig. 4g, lower panels).

### The Y362E mutation increases the number of astral MTs at the old and new SPBs

**Figure 5:**
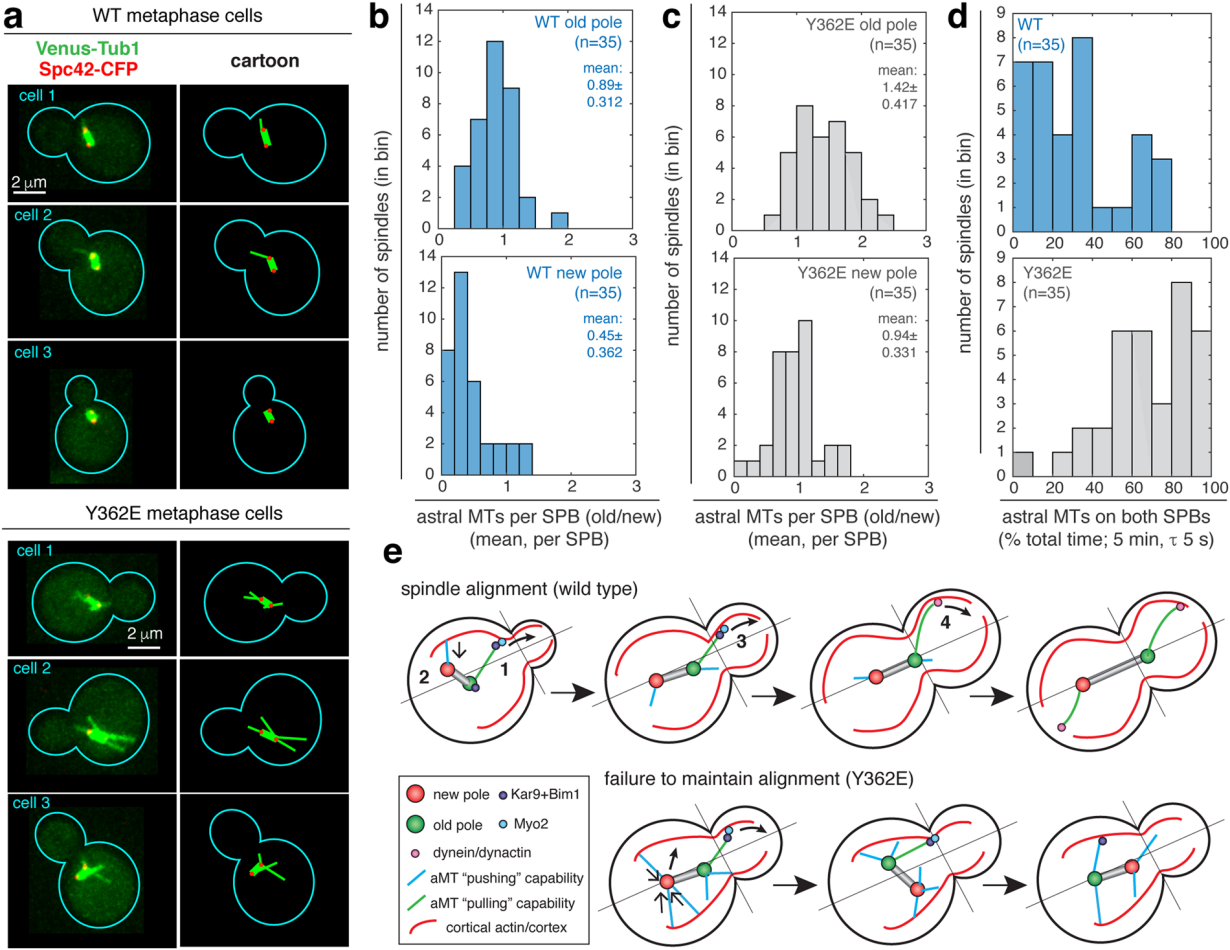
The number of astral MTs associated with old and new SPBs is increased in γtub-Y362E cells. (**a**) Representative metaphase spindles (two SPBs 0.8 to 2 µm apart) in wild-type (WT) and γtub-Y362E cells. MTs are tagged with Venus-Tub1 (shown in green) and SPBs tagged with Spc42-Cerulean (shown in red). Corresponding explanatory schematics for astral MT organization are shown to the right. (**b**,**c**) Astral MT count for old and new SPBs in wild-type (**b**) and γtub-Y362E (**c**) cells. (**d**) Percentage of total time both SPBs have astral MTs in WT and γtub-Y362E cells. (**e**) Proposed mechanism for loss of alignment in γtub-Y362E cells. (1) During normal spindle alignment, Kar9-Bim1 complexes are loaded onto astral MTs and track the plus end. Kar9 binds to the type 5 myosin Myo2 which guides the astral MT plus end to the bud by walking on actin cables that originate at the bud cortex, while astral MTs projecting into the mother cell, regardless of their association with Kar9-Bim complexes, are short lived. (2 and 4) Astral MTs terminating in the bud exert a “pulling” force, via Kar9 or dynein, (3) astral MTs terminating in the mother are capable of “pushing” the spindle away from the mother cell cortex. An increased in the number of astral MTs in γtub-Y362E cells results in uncoordinated pushing and pulling forces (via Kar9 or dynein) at both SPBs, resulting in failure to achieve or maintain spindle alignment.

Forces produced by astral MTs are important for spindle positioning and alignment^44–47^. Thus, we investigated the effect of the γtub-Y362E mutation on the behavior and number of astral MTs in metaphase cells. Representative wild-type and γtub-Y362E cells expressing a Venus-Tub1 (α-tubulin) MT reporter and a Spc42-Cerulean SPB reporter are shown in Fig. 5a and supplemental movies M10–M15. In wild-type cells, pre-anaphase spindles typically have one to two astral MTs (mean of 1.28 ± 0.568), in agreement with published data^48^. Pre-anaphase spindles in the γtub-Y362E mutant had almost two-fold more astral MTs than wild-type (mean of 2.59 ± 0.616, p = 1.5 × 10^−9^).

We next investigated the astral MT number at each SPB. Astral MTs associated with the pre-existing (old) SPB are thought to persist longer due to an increased stability cause by the asymmetric loading of MT + tip proteins, such as Bim1 and Kar9, during spindle alignment^49–51^. The increased stability of astral MTs loaded with + tip proteins likely contributes to the increased number of astral MTs associated with the old SPB (0.89 ± 0.312) relative to the new SPB (0.45 ± 0.362, p = 1.1 × 10^−6^) in wild-type cells (Fig. 5b). However, in γtub-Y362E cells, the number of astral MTs is increased on both old and new SPBs relative to wild-type (Fig. 5c). The number of astral MTs associated with the old SPB increased 1.6-fold (1.42 ± 0.417, p = 1.1 × 10^−6^) and the number of astral MTs associated with the new SPB increased 2-fold (0.94 ± 0.331, p = 1.0 × 10^−6^). Interestingly, despite having increased numbers of astral MTs, only one astral MT entered the bud at a time in Y362E cells, as is seen in wild-type (Supplemental Figure S4). The increase in the number of astral MTs at both old and new SPBs in γtub-Y362E cells results in a significant shift towards symmetry of astral MT occupancy, defined as having at least one astral MT on both SPBs over the five-minute acquisition window (e.g. 80% symmetry of astral MT occupancy indicates the cell has at least one astral MT on both poles for 80% of the acquisition time), and away from the bias of astral MT occupancy asymmetry to the old SPB found in wild-type cells (Fig. 5d, p = 1.3 × 10^−7^). In the γtub-Y362E mutant, 40% of cells (14 of 35 cells) had ≥80% symmetry of astral MT occupancy, a condition that was not observed in wild-type cells (n = 35). Over 80% of γtub-Y362E cells (29 cells) had astral MTs on both SPBs during at least 50% of the five-minute acquisition window, while in wild-type cells, the frequency of symmetry of astral MT occupancy ≥50% was relatively rare (8 cells, 23%). Our analysis suggests that the majority of movements of the spindle during pre-anaphase spindle positioning in γtub-Y362E cells are predominantly influenced by astral MTs associated with both old and new SPBs, rather than by astral MTs associated with the old SPB as in wild-type cells.

## Discussion

In this study, we developed GAMER, a novel SGA-based approach that identifies subtle differences amongst alleles of one gene (γtub-Y445D, γtub-Y362E). In previous SGA screens, the assumption that SGIs are rare events^52^ forms the basis of the analysis, leading rare to be defined contextually amongst the queried double mutants. In contrast, GAMER measures growth rates of individual colonies in combination with mixed effects regression to overcome the limitations that are solved by assuming SGIs are rare, leading to an increased sensitivity in the method. It is ideally suited for discerning the subtle differences between an allelic series and also for identifying SGIs of mutants with subtle growth phenotypes such as the γtub-Y362E mutant. The sensitivity and precision of the GAMER approach enabled the detection of a set of specific SGIs for the γtub-Y362E allele that revealed sensitivity to mutations in genes acting in spindle positioning/alignment and cell polarity, but not to mutations in genes acting in spindle assembly or the spindle assembly checkpoint. These results motivated a quantitative analysis of spindle positioning and alignment; this led to the first instance of evidence that phosphorylation of Y362 may control the number of astral MTs during spindle positioning. Lastly, our method can uniquely be applied to both large-scale and small-scale assays, allowing the functional screening of new candidates using sub-arrays of the SGA. Taken together, and considering its success in detecting subtle differences between γ-tubulin alleles, we believe GAMER to be a useful improvement on the standard SGA method as it facilitates the comparative analysis of an allelic series and aids in the identification of separation-of-function mutants.

GAMER results indicated that the γtub-Y445D and γtub-Y362E alleles perturb different aspects of γ-tubulin’s function, with the former acting in spindle assembly as previously described^17^, and the latter involved in spindle alignment relative to the bud neck plane. Our high-throughput GAMER results recapitulate previous SGIs identified by tetrad dissection^17,24^ and the identified γtub4-Y445D SGIs are consistent with a previously reported decrease in chromosome transmission fidelity in this mutant^20^. We confirmed the latter result by quantitatively measuring the ability of a metaphase spindle to reach the state of perfect alignment in wild-type, the γtub-Y362E mutant, and spindle positioning and alignment mutants kar9∆ and dyn1∆. Unexpectedly, we found that γtub-Y362E cells have an increased number of astral MTs on both old and new SPBs, which reduces the asymmetry of astral MT pole occupancy that is normally biased to the old SPB relative to the new SPB. The increase in astral MT symmetry is correlated with an increase in the frequency of spindle misalignment independent of spindle length.

GAMER analysis combined with analysis of intra-molecular mutations provided new insights into the role of S360 and Y445 phosphorylation in spindle assembly. Mutations that substitute negatively charged amino acids (D/E) at S360 and Y445 decrease spindle stability (^17,19^ and this study). This instability is not due to a lack of spindle MTs, but instead arises from a defect in the organization of these MTs, in particular the formation of a stabilizing core bundle of anti-parallel inter-polar MTs, which is promoted in the γtub-S360A mutant and defective in the γtub-S360D mutant^17,19^. The ase1∆ and slk19∆ SGIs with the γtub-Y445D allele are consistent with a similar defect in the function of the inter-polar MTs of the spindle. The phosphorylation state of S360 and Y445 may be involved in tuning the architecture of the spindle MTs, with phosphorylation state either promoting MT cross-linking (S360 and/or Y445 unphosphorylated) or preventing excessive or inappropriate MT cross-linking (S360 and/or Y445 phosphorylated). Despite their similar phenotypic output of spindle instability, the lethality of the intra-molecular γtub-S360A-Y445D mutant suggests that phosphorylation of S360 and Y445 are likely to be independent events during spindle assembly.

In budding yeast, there are two pathways that can execute pre-anaphase spindle positioning and alignment: the Kar9/Bim1/Myo2 pathway (APC/EB1/Myosin V in metazoans) and the dynein pathway, both of which act by applying a pulling force on astral MTs and, as a result, on the spindle as well^53–56^. We investigated both of these pathways’ role in the achievement of perfect alignment (where the spindle must translocate towards the bud neck, and align with the old SPB proximal to the bud neck plane). The process of spindle translocation and alignment begins in early metaphase, and in the absence of Kar9, short spindles (<2 µm in length) fail to achieve perfect alignment. We found that spindles in kar9Δ cells are frequently oriented parallel to the bud neck plane, consistent with previous studies demonstrating that the kar9Δ mutant exhibits defects in alignment as well as nuclear migration^54,57^. Deletion of dynein did not affect spindle translocation to the bud neck plane, but resulted in an increase in time points where the new SPB is proximal to the bud neck (incorrect spindle alignment). Our results confirm that Kar9 is the dominant pathway for early spindle positioning and alignment.

Our analysis of spindle alignment in the presence of the Y362E mutant combined with dyn1∆ or kar9Δ mutations reveals that both pathways depend on astral MT asymmetry to achieve perfect alignment (Fig. 4e). In the absence of Kar9, the γtub-Y362E mutation enhances misalignment such that spindles appear to explore many more orientations; this observation suggests that dynein may be sensitive to the increase in astral MT number at both old and new SPBs, and/or symmetry of pole occupancy resulting from the γtub-Y362E mutation. This would be unexpected, as the asymmetry of dynein to the astral MTs of the old SPB in late-metaphase has previously been shown to be independent of astral MT number and symmetry^58^. The increase in astral MTs on both SPBs therefore may lead to an increase in pushing forces applied to both old and new SPB of the spindle that arise from the polymerization of astral MTs rather than Kar9 or dynein. When the plus ends of astral MTs come into contact with the cortex of the mother cell, the MT exerts a pushing force on the SPB, causing it to displace^55^; astral MT pushing forces are also observed in G1 cells, prior to SPB seperation^51^. Pushing forces applied by astral MTs do not interfere with spindle positioning at the bud neck^59^ if properly balanced by astral MTs that exert pulling force on the old SPB.

Kar9 and dynein can both pull astral MT plus ends into the bud through their association with cortical actin. Due to the asymmetric localization of Kar9 and dynein, pulling forces are almost exclusively biased to the old SPB in wild-type cells. Pulling forces are usually restricted to one bud-directed astral MT at a time (supplemental Fig. S4); this is likely why Kar9-FP is typically only seen at one focus at a time^44,49,54^. Every astral MT has the potential to apply either a pulling force (bud-directed) or a pushing force (mother-directed). If there is an increase in the number of astral MTs at both SPBs (i.e. a shift towards astral MT occupancy symmetry), as is seen in the γtub-Y362E mutant, an increase in the frequency of pushing forces by astral MT at both SPBs will compete with pulling forces that are biased to the astral MTs of one SPB (Fig. 5e). Interestingly, wild-type and γtub-Y362E cells have at most a single bud-directed astral MT at any time (supplementary Fig. S4). This suggests that the surplus of astral MTs projecting into the mother cell in the γtub-Y362E mutant are applying pushing forces. As a result, γtub-Y362E cells must contend with the potential for pushing and pulling forces applied to the old pole. Taken together, these consequences are expected to produce spindle movements that are decoupled from the polarity axis of the cell that we observe in γtub-Y362E cells.

γ-Tubulin phospho-regulation appears to contribute to both spindle assembly and alignment/positioning (^17,19^ and this study), processes that occur in the nucleus and cytoplasm, respectively. Given the unaltered spindle stability as well as ~2-fold increase in astral MTs, we speculate Y362 phosphorylation occurs in the cytoplasmic compartment of the cell. As well, we suggest that S360 and Y445 phosphorylation occurs in the nucleus and mainly affects those MTs and their organization. Phosphorylation may also be restricted to a sub-population of γ-TuRCs in both compartments. Kollman *et al*. proposed that S360 and Y362, which are located on the inner side of the γ-TuRC, can only be accessed by a kinase when the γ-TuRC is not occupied by a MT^15^. Phosphorylation of Y362 is therefore likely to occur prior to nucleation of a MT by the γ-TuRC.

The appearance of metaphase spindles in γtub-Y362E cells is similar to metaphase spindles in wild-type cells (Fig. 5a and supplemental movies M10–M15) and it is unclear whether the number of spindle MTs is increased by the γtub-Y362E mutation. The budding yeast kinetochore binds a single MT, and thus surplus spindle MTs would increase the number of anti-parallel cross-linked inter-polar MTs which has been shown to not significantly alter spindle function^19^. In wild-type cells, the formation of inter-polar MTs does not compete with the capture of kinetochores by free MT plus ends^60^, and cross-linking proteins such as Ase1 or Cin8 may limit the number of stable inter-polar MTs that can be formed^19^. Spindle MTs not bound to a kinetochore or another MT are relatively unstable, and for this reason the formation of surplus spindle MTs during spindle assembly may not result in major changes in spindle architecture.

The Y362E mutation introduces a negatively charged amino acid (E) in place of a tyrosine known to be phosphorylated *in vivo*^16^, and suggests that phosphorylation of Y362 is likely to increase the number of astral MTs, either by stabilizing and thereby increasing the lifetime of astral MTs or by favoring the formation of astral MTs. Surprisingly, the increase in symmetry of astral MT occupancy we observed in γtub-Y362E cells appears to not block translocation of the spindle to the bud neck in the absence of dynein, suggesting that Kar9 is functional even when astral MTs are present on both SPBs^49,57,61^. Furthermore, while both old and new SPBs are associated with more astral MTs in the majority of spindles in γtub-Y362E cells, the number of astral MTs associated with the old SPB (1.42 ± 0.412) is greater than that of the new SPB (0.94 ± 0.331, p = 8.2 × 10^−6^); in fact, the difference in astral MT number between the old and new poles does not change significantly between wild type and γtub-Y362E (0.44 ± 0.081 and 0.48 ± 0.090, respectively; p = 0.92). This suggests that although astral MT occupancy becomes symmetric, factors contributing to differences in astral MT number remain active in γtub-Y362E cells. Regardless of the mechanism by which the number of astral MTs is increased, our analysis of the γtub-Y362E mutant suggests that γ-tubulin contributes to determining the number of astral MTs associated with the spindle during metaphase, and consequently contributes to spindle alignment. Our findings raise several fascinating questions for future studies. We speculate that phosphorylation of Y362E may increase the number of astral MTs associated with the old SPB, and increase the efficiency of pre-anaphase spindle alignment by Kar9. Alternatively, phosphorylation of Y362 may promote astral MT symmetry and the efficiency of dynein in maintaining the position of the SPBs in the mother and bud compartments. The 2-fold increase in the number of astral MTs associated with the new SPB, which frequently lacks astral MTs in wild-type cells, opens the possibility that phosphorylation of Y362 may contribute to γ-TURC activation, which has previously been shown to increase nucleation by ~2-fold^15^. Given the evolutionary conservation of Y362 across eukaryotes, phosphorylation could be part of a general mechanism for control of microtubule number.

## Methods

### Strains, Plasmids, Genetic Manipulation and Growing Conditions

All yeast strains are derivatives of BY4741^62^ and are listed in Supplemental Table I. All plasmids used in this study are listed in Supplemental Table II. Point mutations (*Y362E*, *Y445D*, etc.) were generated on plasmids using USER^TM^ cloning^63^ followed by sequencing to confirm error free introduction of the desired mutation. PCR-based methods were used to integrate point mutations and fluorophore tags into the native gene locus on the chromosome under their endogenous promoters^64^. Venus-Tub1 was made by integrating pHIS3p:Venus-Tub1 + 3′UTR::HIS3 (gift from Wei-Lih Lee, Addgene plasmid # 50656)^65,66^ into the knocked out his3Δ1 locus of BY4741, with the Venus-Tub1 fusion expressed under the native *HIS3* promoter. Yeast strains were grown in YEPD medium^67^ at 25 °C unless otherwise stated. For fluorescence live-cell microscopy, cells were grown in synthetic complete (SC) medium^68^. Bacterial cells used from cloning were grown at 37 °C in LB medium supplemented with 100 μg/mL of carbenicillin.

### Live cell microscopy

Yeast cells expressing Spc42-Cerulean were grown to log phase in SC medium supplemented with 2 mM of ascorbate, and imaged at 25 °C on a custom-built spinning-disk confocal microscope as previously described^19^. Briefly, the following components were installed on a Leica DM6000 inverted microscope: a 100x/1.46 numerical aperture plan apochromatic objective, an XY stage with a Z top piezo (Applied Scientific Instrumentation), a Borealis head (a Quorum conversion of a Yokogawa QLC-100). Solid state lasers (446 and 515 nm) with an emission filter wheel were used for multicolor imaging. A Hamamatsu ImagEM EM-CCD camera was used for detection. MetaMorph (Molecular Devices) was used for image acquisition. Images were collected in a streaming regime as Z-stacks (200 or 300 nm Z-steps across 31 or 30 focal planes, respectively) with an integration time of 50 ms per focal plane. For spindle dynamics and alignment studies, cells were imaged over ten minutes with a ten second time step between stack acquisitions. For imaging astral MTs in live cells, strains containing Venus-Tub1 were imaged over five minutes with five second time step between stack acquisitions (the Spc42-Cerulean pole reporter was collected with ten second time steps in these strains).

### Analysis of spindle stability, alignment, positioning and orientation

Pole-tracking was implemented using MatLab (Mathworks) as previously described^19^. Briefly, SPBs were fit to 3-dimensional gaussians and tracked by nearest neighbor analysis. Spindle stability was measured by computing the standard deviation of the instantaneous spindle lengths (referred to as spindle fluctuations) of each cell over the ten-minute time course. For spindle positioning and alignment, the bud neck was defined by manually drawing a line through the neck in the brightfield image. Spindle positioning at the bud neck was measured by calculating the mean distance between the SPB proximal to the bud and the bud neck. This distance was normalized to the length of the mother to adjust for minor differences in the size of the mother compartment. One-dimensionally projected spindle length was calculated by projecting the SPB coordinates onto the mother-daughter axis, perpendicular to the bud neck plane. Spindle orientation was assessed by first assigning the SPBs as either old or new, based on the mean integrated intensity of Spc42-Cerulean over the ten minute time course, with the brighter SPB identified as the old SPB^19^. A spindle was considered mis-oriented if the SPB proximal to the bud neck had a lower mean integrated intensity (i.e. was the ‘new’ SPB) than the distal pole. Spindle alignment heatmaps were generated first as bivariate histograms of 30 × 30 bins and then smoothed with bicubic interpolation to give 180 × 180 bins. The color indicates the normalized frequency of data points in each bin across all analyzed cells; the maximum normalized frequency (dark red) indicates all bins with a normalized frequency ≥0.015.

### Analysis of astral MTs

Wild-type and γtub-Y362E cells with astral MTs labeled using Venus-Tub1 were analyzed using Fiji^69^. Astral MTs projecting from each SPB (labeled with Spc42-Cerulean) were detected in maximum projections and counted manually in all time points (61 time points per cell over five minutes, with a time step of five seconds). Astral MTs were only counted once they were resolvable from the central spindle and could be observed for at least two-time points. This dataset was used to compute the mean number of astral MTs per spindle, the mean number of astral MT per SPB, and the number of time points where both SPBs had at least one astral MT during the five-minute acquisition. Intensity of astral MTs was not considered, i.e. we did not discriminate between bundled astral MTs which would appear brighter than a single astral MT, therefore, these data may underestimate the number of astral MTs per spindle/SPB.

### Statistics used for Image Analysis

Distributions were tested for normality using a one-sample Kolmogorov-Smirnov test. To test for differences in spindle fluctuations, the variance of changes in spindle length (i.e. between two neighboring time points) was assessed between strains using a Bartlett’s test. Distributions for normalized mean distance from the neck were assessed using the Kruskal-Wallis test followed by a Dunn-Šidák correction. Distributions for the mean number of astral MTs per spindle and for the percentage of total time both poles had astral MTs were assessed for differences using the Wilcoxon rank sum test/Mann-Whitney U-test.

### Synthetic Genetic Array

Strains undergoing the SGA process were iteratively grown to select for double mutants using previously described media^27^ with the aid of a Beckman Coulter Biomek robot. In order to measure the growth rates, an additional robotics platform based on Beckman Coulter’s robotic Orca Arm photographs each final selection plate every two hours for 72 hours.

### SGA Image Processing

After data acquisition, the colony densities were extracted from each image as the integrated intensity. To maximize colony detection, a computationally generated grid was overlaid on each plate, where each grid tile contained at most a single colony and was analyzed from the last time point back to the first time point. Each grid tile was analyzed for the presence of a colony. A watershed algorithm was then applied to separate the colony from the background, and density was measured as the integrated intensity over the background of these pixels.

To generate a grid which does not intersect a colony, we first identified colonies using a coarse-grained method based on the circular Hough transform. Since the colonies are pinned in a regular pattern, circular objects that are either too close or too far from one another are removed. The grid lines are fit to these objects such that they do not intersect with the objects and follow the regular pattern of the pin tool resulting in grid tiles containing at most one colony. When an insufficient number of objects was found for grid fitting (less than 100), the grid lines from the later time point were used.

In order to measure the fitness of a colony, the densities were fit to a Gompertz growth equation of the form:

1 $$Density\,(time)=A\ast \exp (B\ast \exp (-GR\ast time))$$

where *A* is the maximum density of the colony, *B* is proportional to the initial size at time 0, *exp* is the exponential function with base e, *GR* is the growth rate and *time* is the elapsed time since the first data point. Here, the growth rate parameter is akin to the doubling time of a single cell and represents the health of the colony. We then normalized the colony densities such that

2 $$Density\,(time)=1\ast \exp (B\ast \exp (-GR\ast time))$$

Then, we applied a double logarithm to transform the Gompertz equation into a simple linear equation:

3 $$\mathrm{log}(\mathrm{log}(Density(time))=\,\mathrm{log}(B)-GR\ast time$$

which is of the form: *y* = *b* + *m* * *x*. Since the parameter of interest is the growth rate, we re-centered time such that log (B) = 0, giving the equation:

4 $$\mathrm{log}(\mathrm{log}(Density(time^{\prime} ))=-GR\ast time^{\prime} $$

where time’ is the shifted time.

### GAMER Mathematical Model

We defined the competitive fitness of strain *i* as: *F*_*i*_ = *GR*_*i*_/*GR*_*WT*_ where *GR*_*i*_ is the growth rate of strain *i* and *GR*_*WT*_ is the growth rate of a wild-type reference. A true genetic interaction is inferred when the fitness of the double mutant is significantly different from its expected fitness (defined as the product of the fitness of the individual mutations)^33,70–72^:

5 $${F}_{ij}={F}_{i}\ast {F}_{j}$$

where *F*_*ij*_ is the fitness of the double mutant with mutations *i* and *j*.

This definition of the expected fitness of the double mutant is appropriately modeled by a single linear mixed-effects model. This allows us to model the growth rates of each colony of the four genotypes (WT, *i*, *j*, *ij*) using two categorical variables. The only assumption of the model is that all the yeast mutants we screened are derived from a common background strain. This allowed us to assume that any observable growth defects in the mutants (both the single and double mutants) can be attributed to the mutation(s).

For the single mutant *i*, the growth rate was modeled as: *GR*_*i*_ = *GR*_*WT*_ + *d*_*i*_, where *d*_*i*_ is the measurable defect in the growth rate associated to the mutation relative to the common background strain. In the case of the double mutant, the growth rate was modelled as *GR*_*ij*_ = *GR*_*WT*_ + *d*_*i*_ + *d*_*j*_ + *d*_*ij*_, where *d*_*ij*_ is the measurable growth defect present only in the double mutant. The expected value of *d*_*ij*_ can be determined by substituting the fitness terms in the expected fitness equation with the growth rate terms:

6 $$\frac{G{R}_{ij}}{G{R}_{WT}}=\frac{G{R}_{i}}{G{R}_{WT}}\ast \frac{G{R}_{j}}{G{R}_{WT}}$$

7 $$G{R}_{ij}=\frac{G{R}_{i}\ast G{R}_{j}}{G{R}_{WT}}$$

8 $$G{R}_{WT}+\,{d}_{i}+{d}_{j}+{d}_{ij}=\frac{(G{R}_{WT}+{d}_{i})\ast (G{R}_{WT}+\,{d}_{j})}{G{R}_{WT}}$$

9 $$G{{R}_{WT}}^{2}+\,G{R}_{WT}\ast \,{d}_{i}+G{R}_{WT}\ast {d}_{j}+G{R}_{WT}\ast {d}_{ij}=G{{R}_{WT}}^{2}+G{R}_{WT}\ast {d}_{i}+G{R}_{WT}\ast {d}_{j}+{d}_{i}\ast {d}_{j}$$

10 $$G{R}_{WT}\ast {d}_{ij}={d}_{i}\ast {d}_{j}$$

11 $${d}_{ij}=\frac{{d}_{i}\ast {d}_{j}}{G{R}_{WT}}$$

We therefore find the expected value for the defect to be:

12 $${d}_{ij}=\frac{{d}_{i}\ast {d}_{j}}{G{R}_{WT}}$$

From this model, a genetic interaction is inferred between the two mutations when the experimentally measured value for *d*_*ij*_ is significantly different from the expected value. The GAMER scores were calculated as:

13 $$GAMER\,score={d}_{i{j}_{expected}}-{d}_{i{j}_{observed}}$$

To calculate p-values, we bootstrap the model 20,000 times and calculate the probability of observing a more extreme value than the expected *d*_*ij*_. Finally, we correct the p-values for multiple hypothesis testing by the false discovery rate.

### Code and data availability

All strains, code, data and analysis is available upon request.

## Electronic supplementary material

Supplemental Tables and Figures

movie M1

movie M2

movie M3

movie M4

movie M5

movie M6

movie M7

movie M8

movie M9

movie M10

movie M11

movie M12

movie M13

movie M14

movie M15

dataset1
