# Author Correction: Interrogation of γ-tubulin alleles using high-resolution fitness measurements reveals a distinct cytoplasmic function in spindle alignment

**DOI:** 10.1038/s41598-018-19512-w

**Published:** 2018-02-01

**Authors:** Kristian Shulist, Eric Yen, Susanne Kaitna, Allen Leary, Alexandra Decterov, Debarun Gupta, Jackie Vogel

**Affiliations:** Department of Biology, McGill University, 3649 Promenade Sir William Osler, Montreal, Quebec, H3G 0B1 Canada

Correction to: *Scientific Reports* 10.1038/s41598-017-11789-7, published online 12 September 2017

The Acknowledgements section in this Article is incomplete.

“The authors thank current and past members of the Vogel lab for stimulating discussions during this work, Brian Leung (McGill Biology) for input during the development of GAMER, and Elke Küster-Schöck and Guillaume Lesage for robotics support.”

should read:

“The authors thank current and past members of the Vogel lab for stimulating discussions during this work, Brian Leung (McGill Biology) for input during the development of GAMER, and Elke Küster-Schöck and Guillaume Lesage for robotics support. The research was supported by a fellowship from the Natural Sciences and Engineering Research Council awarded to KS and operating grants from the Natural Sciences and Engineering Research Council (262246), the Canadian Institutes of Health Research (123335) and the Fonds Nature et technologies (191128) awarded to JV.”

