# Supplementary material for: Interrogation of γ-tubulin alleles using high-resolution fitness measurements reveals a distinct cytoplasmic function in spindle alignment
Source: Sci Rep. 2017 Sep 12;7:11398. doi: 10.1038/s41598-017-11789-7 (PMC5595808; doi:10.1038/s41598-017-11789-7)
Supplement: Supplementary file 1 — Supplemental Tables and Figures [file 41598_2017_11789_MOESM1_ESM.pdf]

Supplemental Tables and Figures for

# Interrogation of $\gamma$-tubulin alleles using high-resolution fitness measurements reveals a distinct cytoplasmic function in spindle alignment

Kristian Shulist[1,2], Eric Yen[1,2], Susanne Kaitna[1], Allen Leary[1], Alexandra Decterov[1], Debarun Gupta[1] and Jackie Vogel[1,3]

## Supplemental Tables

## Supplemental Table I. Yeast strains used in this study

| Yeast Strain | Genotype | Notes | Reference |
|---|---|---|---|
| YV838, YV856, YV2175, YV2176 | *SPC42-cerulean-HYGB; MATa; his3Δ1; leu2Δ0; ura3Δ0; met15Δ0* | wild-type; isolate 1, 2, 3, 4 | Nazarova, et al., 2013 and this study. |
| YV2275, YV2276, YV2464 | *tub4-Y362E-NAT; SPC42-cerulean-HYGB; MATa; his3Δ1; leu2Δ0; ura3Δ0; met15Δ0* | γ-tub-Y362E; isolate 1, 2, 3 | This study. |
| YV2098, YV2361, YV2362 | *tub4-Y445D-NAT; SPC42-cerulean-HYGB; MATa; his3Δ1; leu2Δ0; ura3Δ0; met15Δ0* | γ-tub-Y445D; isolate 1, 2, 3 | This study. |
| YV2230, YV2231, YV2232 | *kar9Δ::KAN; SPC42-cerulean-HYGB; MATa; his3Δ1; leu2Δ0; ura3Δ0; met15Δ0* | kar9Δ; isolate 1, 2, 3 | This study. |
| YV2235, YV2236, YV2237 | *dyn1Δ::KAN; SPC42-cerulean-HYGB; MATa; his3Δ1; leu2Δ0; ura3Δ0; met15Δ0* | dyn1Δ; isolate 1, 2, 3 | This study. |
| YV2331, YV2332, YV2333 | *tub4-Y362E-NAT; kar9Δ::KAN; SPC42-cerulean-HYGB; MATa; his3Δ1; leu2Δ0; ura3Δ0; met15Δ0* | γ-tub-Y362E; kar9Δ; isolate 1, 2, 3 | This study. |
| YV2492, YV2493 | *tub4-Y362E-NAT; dyn1Δ::KAN; SPC42-cerulean-HYGB; MATa; his3Δ1; leu2Δ0; ura3Δ0; met15Δ0* | γ-tub-Y362E; dyn1Δ; isolate 1, 2 | This study. |
| YV2500, YV2501, YV2502 | *HIS3::venus-TUB1; SPC42-cerulean-HYGB; MATa; his3Δ1; leu2Δ0; ura3Δ0; met15Δ0* | wild-type; isolate 1, 2, 3 | This study. |
| YV2509, YV2510, YV2511 | *tub4-Y362E-NAT, HIS3::venus-TUB1; SPC42-cerulean-HYGB; MATa; his3Δ1; leu2Δ0; ura3Δ0; met15Δ0* | γ-tub-Y362E; isolate 1, 2, 3 | This study. |

Supplemental Table II. Plasmids used in this study

| Plasmid | Description | Reference |
|---|---|---|
| pJV0372 | pBKS-*tub4*-NAT | This study. |
| pJV0373 | pBKS-*tub4-Y362E*-NAT | This study. |
| pJV0374 | pBKS-*tub4-Y445D*-NAT | This study. |
| Addgene #50656 | pHIS3p:Venus-Tub1+3'UTR::HIS3 | Markus et al., 2015 |

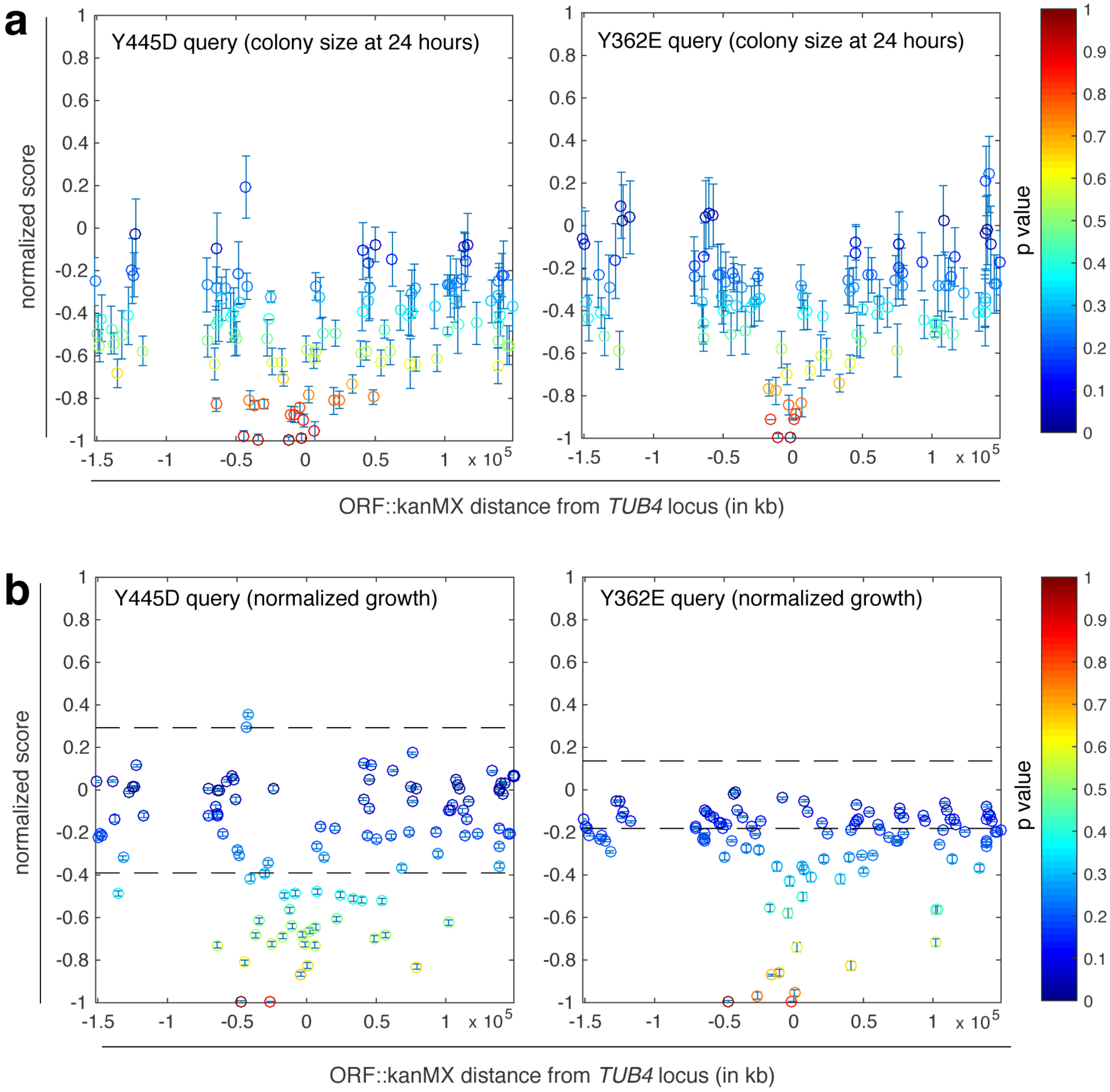


**Figure S1 | Growth rates reflect strain fitness independently of initial conditions.** Linkage group analysis for Y445D and Y362E alleles based on colony size (a) and measured growth rates (b). A linkage group extending ~150 kb (50 cM) on either side of the *TUB4* locus is detected for the Y445D and Y362E alleles using both methods. Colony sizes at 24 hours have a large variance that increases with distance. This variance arises from initial conditions and experimental procedures, and must be corrected in post-processing. In contrast, measured growth rates for double mutants have increased precision and accuracy as a consequence of relatively uniform growth rates across replicates and duplicates.

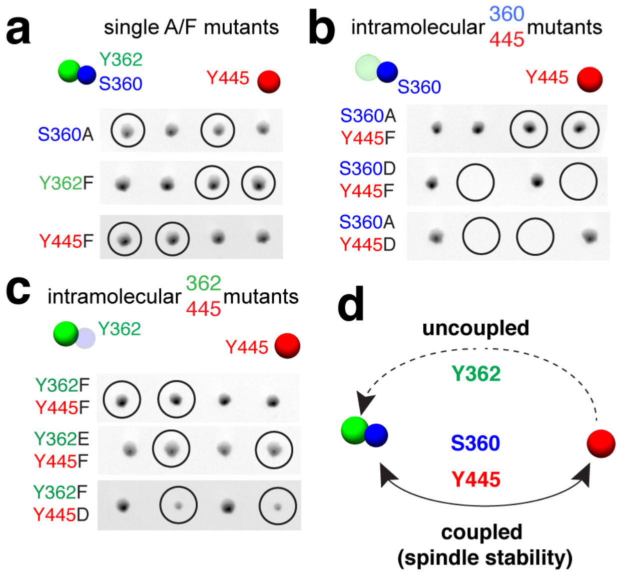


**Figure S2 | Y362E is a separation of function allele.** Meiotic outcomes of (a) single phosphor-inhibiting mutants and intramolecular phosphor-mutants between (b) S360 and Y445 and (c) Y362 and Y445. While individual phosphor-inhibiting mutations S360A, Y362F and Y445F have no detectable growth phenotype, lethality is observed when combined with phosphor-mimetic mutations S360D or Y445D. Intramolecular co-lethality suggests functional coupling between S360 and Y445. A weak growth defect is observed between the Y362F and Y445D mutant. (d) residues S360 and Y445 contribute to spindle stability, while Y362 contributes to spindle positioning.

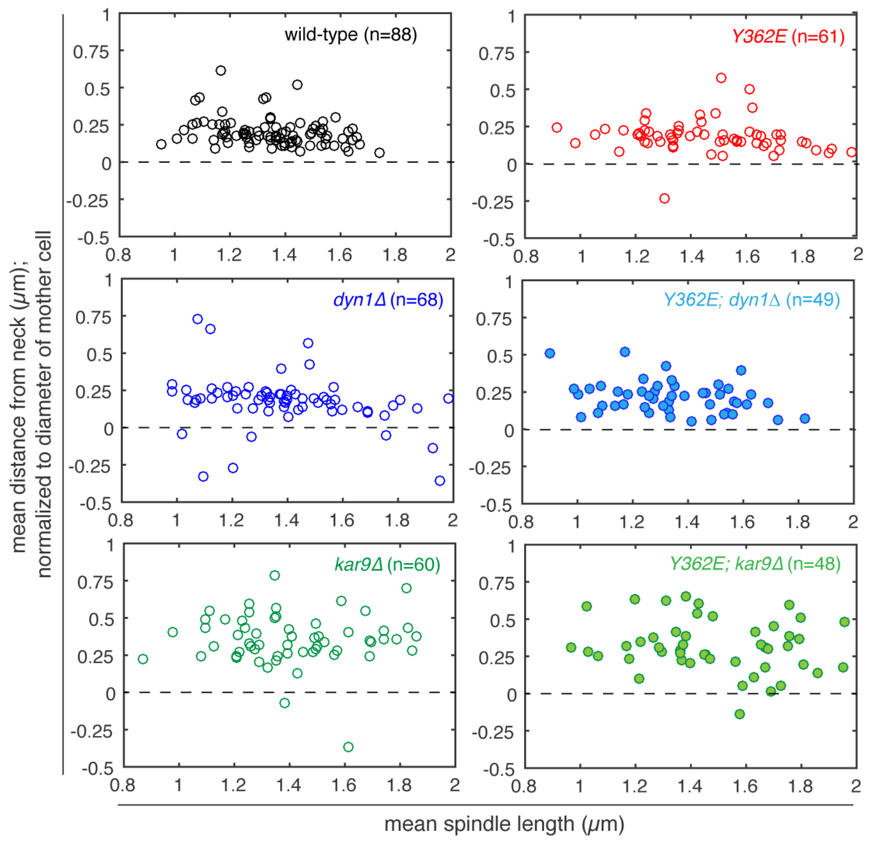


**Figure S3 | γTub-Y362E does not perturb spindle positioning at the bud neck.** Distance from the proximal pole to the bud neck, normalized to the initial length of the mother cell, plotted as a function of mean spindle length for wild-type cells and γtub-Y362E, kar9Δ, dyn1Δ single mutants and γ-tubY362E; kar9Δ and γtub-Y362E; dyn1Δ double mutants.

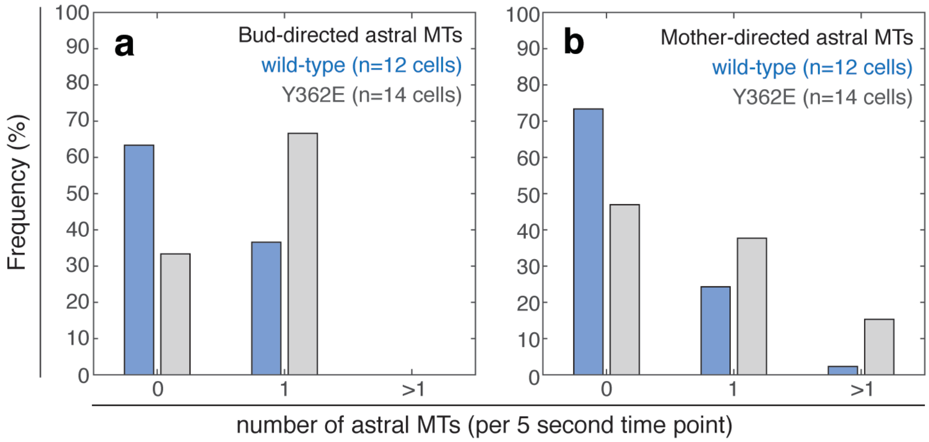


**Figure S4 | A single astral MT enters the bud in both wild-type and γtub-Y362E cells.** The number of bud-directed astral MTs never exceeds one per 5 second time point in both wild-type (n = 12 cells) and γtub-Y362E (n = 14 cells). Frequency of time points in which cells had 0, 1, or >1 (a) bud-directed or (b) mother-directed astral MT(s).
